# Commentary: Leveraging big data in health care and public health for AI driven talent development in rural areas

**DOI:** 10.3389/fpubh.2025.1685569

**Published:** 2025-09-30

**Authors:** R. Sujithra, V. Bini Marin, B. Sreya

**Affiliations:** School of Enterpreneurship and Management, Joy University, Vaddakkankulam, Tamil Nadu, India

**Keywords:** big data in health care, public health talent development, AI in rural health systems, healthcare workforce optimisation, health policy

## 1 Introduction

Zhou et al. (1) present the Transformer-driven Adaptive Talent Engagement Model (ATEM), an innovative framework addressing the long-standing challenge of attracting and retaining healthcare professionals in rural areas. By integrating big data analytics, natural language processing, and adaptive incentive strategies, ATEM tackles a multifaceted issue that has constrained rural health systems for decades. This is especially relevant given the urgent need to bridge urban–rural healthcare disparities, where shortages of qualified professionals contribute to preventable morbidity and mortality. ATEM combines advanced AI-driven matching algorithms with strategic incentive allocation, leveraging large datasets and dynamic feedback loops to respond to evolving workforce demands and professional motivations. Benchmarking against existing models shows notable improvements in recommendation accuracy, candidate–role matching, and retention forecasting, indicating significant potential for rural healthcare workforce planning. This commentary recognizes the technical innovation and conceptual strength of ATEM while emphasizing the importance of real-world validation.

## 2 Strengths and contributions

ATEM's integration of socio-economic, motivational, and community-based parameters into a unified AI system reflects a holistic approach, consistent with evidence that effective rural health workforce retention requires both financial and non-financial incentives (2). By modeling candidate–role alignment for optimal placement (3), incorporating adaptive feedback mechanisms for continuous strategy refinement, and leveraging scalable pre-trained Transformer models tailored to rural healthcare needs (4), ATEM demonstrates both technical sophistication and practical adaptability. Its incentive allocation strategies, informed by behavioral patterns and contextual realities, enhance its ability to address diverse retention challenges. Collectively, these features position ATEM as a forward-looking, versatile tool for sustainable rural healthcare workforce development.

## 3 Critical perspective: the evidence gap

While the model performs strongly in recommendation accuracy and retention forecasting using benchmark datasets such as MovieLens, Gowalla, Foursquare, and KuaiRec (5, 6), these datasets are not healthcare-specific. Although computationally robust, they fail to capture rural healthcare complexities, including professional isolation, cultural integration, and disparities in access to continuing education (7). As a result, real-world deployment may reveal challenges not evident in generic datasets (8). Addressing this gap, the authors plan to collaborate with healthcare institutions to collect domain-specific datasets—an essential step for assessing the model's practical relevance and applicability to rural workforce challenges (9).

Beyond data limitations, rural areas face several structural barriers that complicate AI deployment (10). Limited internet connectivity often restricts the effective use of cloud-based AI tools, while gaps in digital literacy among both healthcare staff and local communities hinder adoption. For instance, community clinics in parts of sub-Saharan Africa have resisted telemedicine platforms due to staff discomfort with digital interfaces, despite their potential benefits. Similarly, in rural India, inadequate training in digital health literacy among healthcare workers has slowed the integration of AI-enabled decision-support systems. These examples illustrate how infrastructural constraints and human-capital limitations remain significant challenges to the real-world implementation of AI in rural healthcare.

## 4 Implications for public health policy and practice

If validated in real-world contexts, ATEM could serve as a valuable decision-support tool for policymakers by guiding targeted recruitment campaigns (11), enabling efficient allocation of incentives within budgetary constraints, and facilitating proactive engagement monitoring to reduce early attrition (12). By combining local socio-economic indicators with individual professional preferences, the framework offers a robust, data-driven foundation for workforce planning that can strengthen service delivery, improve health equity, and reduce disparities in access to care in underserved communities (13). Successful implementation of AI in rural settings also requires embedding robust ethical and community-centered safeguards into deployment strategies (14). This involves establishing strict data privacy protocols to protect sensitive professional and patient information, conducting routine fairness audits to minimize algorithmic bias, and creating mechanisms for obtaining informed community consent before introducing AI-driven recruitment initiatives. Such measures are essential to ensure transparency, foster trust among rural populations, and align technological innovation with ethical standards and cultural expectations.

## 5 Discussion

ATEM's primary contribution lies in its integration of advanced AI capabilities with the strategic objective of strengthening rural healthcare workforce capacity (1). Achieving real-world success, however, will require addressing three key factors. First, datasets must accurately reflect the realities of rural healthcare. Second, robust ethical safeguards are essential to ensure fairness, transparency, and privacy (15). Third, engagement strategies should be tailored to local traditions, values, and community priorities to ensure cultural relevance and sustainable impact.

The adoption of ATEM in rural settings faces critical hurdles, including limited resources for digital infrastructure, insufficient technical expertise, and skepticism from health workers concerned about automation displacing human judgment. Overcoming these barriers requires robust stakeholder engagement through coordinated efforts with ministries of health, local governments, and NGOs to mobilize resources, align policies, and deliver targeted training. Integrating community leaders into system design and rollout further enhances legitimacy and local adaptation, while NGO partnerships play a pivotal role in bridging gaps in capacity-building and infrastructure (16).

## 6 Conclusion and future research

ATEM represents a technically advanced, conceptually comprehensive approach to rural health talent development. Realizing its full potential will require deployment using authentic rural healthcare datasets to rigorously evaluate predictive accuracy, retention outcomes, and policy implications. Future research should consider longitudinal applications across regions, assess cross-cultural adaptability, and establish robust ethical frameworks for AI-driven recruitment. Moving from computational validation to demonstrated field effectiveness could significantly enhance rural workforce sustainability and contribute to reducing healthcare disparities globally.
